# Shear Wave Ultrasonographic Elastography in Pediatric Spleens and Its Role in Differential Diagnosis

**DOI:** 10.3390/diagnostics14111142

**Published:** 2024-05-30

**Authors:** Turkhun Cetin, Oguzhan Tokur, Hayrunnisa Bekis Bozkurt, Sonay Aydin, Kemal Bugra Memis, Mecit Kantarci

**Affiliations:** 1 Department of Radiology, Erzincan University, Erzincan 24100, Turkey; turkhun.cetin@erzincan.edu.tr (T.C.); sonay.aydin@erzincan.edu.tr (S.A.); kemalbugramemis@gmail.com (K.B.M.); akkanrad@hotmail.com (M.K.); 2 Department of Radiology, Kutahya Health Sciences University, Kutahya 43020, Turkey; 3 Department of Pediatrics, Kafkas University, Kars 36000, Turkey

**Keywords:** childhood, spleen, stiffness, ultrasound, shear wave elastography (SWE)

## Abstract

Shear wave elastography (SWE) has become popular in clinical practice for many diseases. However, there is not adequate research on spleen-related diseases. This study aimed to investigate the potential of quantitative values obtained through SWE in evaluating spleen pathologies in the pediatric population and to demonstrate its performance to differentiate splenomegaly-related diseases. The research group retrospectively included children with pathological diagnoses related to the spleen from November 2016 to April 2021, and they were categorized into three groups, including portal hypertension (PH), benign lymphoid hyperplasia (BLH), and malignant infiltration (MI). Spleen sizes and parenchymal stiffness were also calculated for each group. Subsequently, mean spleen stiffness in each group was compared with normal values within the same age group. In total, 2781 children (1379 children for the study group; 1402 children for the control group) were enrolled in the study. The highest stiffness was observed in the PH group, which is statistically higher than others (*p* < 0.05). Although the mean spleen stiffness in the group with BLH was higher than the control and MI group, the difference was not statistically significant (*p* = 0.08). The mean stiffness in the group with MI was significantly lower than both the control group (*p* = 0.005) and PH (*p* = 0.01). In conclusion, using SWE in the differential diagnosis of etiologies causing splenomegaly could make an important contribution.

## 1. Introduction

During the initial stages of ultrasound elastography, tissues were manually compressed, and the response of tissues to this force was measured to evaluate tissue stiffness, which limited the examination of only superficial tissues [1]. After modern advancements, however, more objective, and quantitative measurements could become possible through modern elastography devices. This has led to their utilization as a part of routine clinical practice, especially for differentiating between malignant and benign lesions in parenchymal tissues, such as the prostate, thyroid, and breast [2,3,4,5,6,7,8].

Shear wave elastography (SWE) is one of the current methods in ultrasound elastography, which is increasingly preferred in diagnosis, providing both quantitative and qualitative values about the elasticity of the related tissue. This method utilizes ultrasound waves to determine the elasticity of tissues and assist in detecting differences among tissues [9]. Transient elastography (TE) is another SWE technique that is mostly used in the liver and spleen. Unlike other SWE methods in which the stimulus is an acoustic push pulse, shear waves are generated by mechanical stimulus in TE. Each method has its advantages and disadvantages, but there are still insufficient studies that have established quality standards for SWE compared to the numerous studies conducted for TE, but both methods have been promising diagnostic methods [10,11,12,13,14,15,16].

The spleen, the largest organ of the immune system, has also become a subject of increasing interest in elastography studies. As a result of this, SWE can be beneficial in differential diagnosis regarding the diseases that affect the spleen. While numerous studies in the literature have examined the relationship between spleen stiffness and the severity of liver fibrosis in patients with portal hypertension, studies focusing on elastography examinations in patients with spleen enlargement due to causes other than portal hypertension are limited in number and have small sample sizes [17,18,19,20,21,22,23,24].

Ultrasound elastography has shown significant advancements in recent years. Quantitative methods, such as transient elastography, acoustic radiation force impulse, and shear wave elastography, have superseded qualitative methods [15,25]. Due to the spleen’s surface location, sonoelastography can provide precise measurements of its hardness with high reliability [26]. Currently, numerous researchers are studying the alterations in spleen elasticity among individuals afflicted with hepatitis B virus or hepatitis C virus, as well as those with liver fibrosis, portal hypertension, esophageal varices, or myelofibrosis [16,18,27,28]. This paper examines the function and present condition of accessible qualitative ultrasound elastography techniques, encompassing recent developments in the assessment of spleen stiffness and its clinical usefulness. Research findings indicate that there is a direct relationship between the stiffness of the spleen and the presence of liver fibrosis. This information is valuable in assessing the extent of fibrosis according to the METAVIR scoring system [29]. Patients with hepatitis B virus or hepatitis C virus see an increase in spleen stiffness, even if the elasticity of the liver remains unchanged. Moreover, it is valuable in the diagnosis of portal hypertension or the prediction of the presence of esophageal varices [18]. Furthermore, spleen sonoelastography can assist in the identification of suitable candidates for liver transplantation and in determining the optimal approach for portal vein reconstruction in patients with biliary atresia following Kasai portoenterostomy [30]. Spleen stiffness in myelofibrosis is directly related to bone marrow fibrosis and can be utilized as a measure to evaluate the effectiveness of treatment [31]. Spleen sonoelastography is additionally valuable for monitoring the functionality of transjugular intrahepatic portosystemic shunts [26].

The aim of this study was to demonstrate the potential of quantitative data obtained through SWE in the evaluation of spleen pathologies in the pediatric population. As a secondary goal, the study aimed to demonstrate the diagnostic efficacy of SWE in distinguishing diseases that lead to splenomegaly, including benign causes such as lymphoid hyperplasia, malignant infiltrative diseases, and portal hypertension.

## 2. Material and Methods

### 2.1. Patients

This study was conducted after obtaining approval from the ethics committee of our tertiary health care hospital (No. 80576354-050-99/169, 05.2019). It included a retrospective analysis of pediatric patients who were referred to the radiology department for abdominal ultrasonography (US) over a period of 5 years from November 2016 to April 2021, did not meet the exclusion criteria, and had pathological diagnoses related to the spleen. Since our study was conducted retrospectively over a relatively long period, patients were selected from electronic records among those who had received a definitive diagnosis through anamnesis, physical examination, laboratory tests, and imaging, and they were divided into three groups (benign lymphoid hyperplasia, malignant infiltrative diseases, and portal hypertension) accordingly. Patients with a history of acute splenic injury, splenic laceration, splenic surgery, previous treatment with non-selective beta-blockers, shunt placement, band ligation, liver transplantation, overt hepatic encephalopathy, intrahepatic or extrahepatic malignancies other than lymphoid malignant infiltration; portal vein thrombosis or cavernous transformation; the presence of other types of chronic liver disease, including autoimmune hepatitis; and any other viral hepatitis or alcohol-related disease were excluded from the study (Table 1). Both written and verbal information were presented to the parents of the participants prior to the examination, and their consent was obtained in writing.

### 2.2. Methods

**Figure 1:**
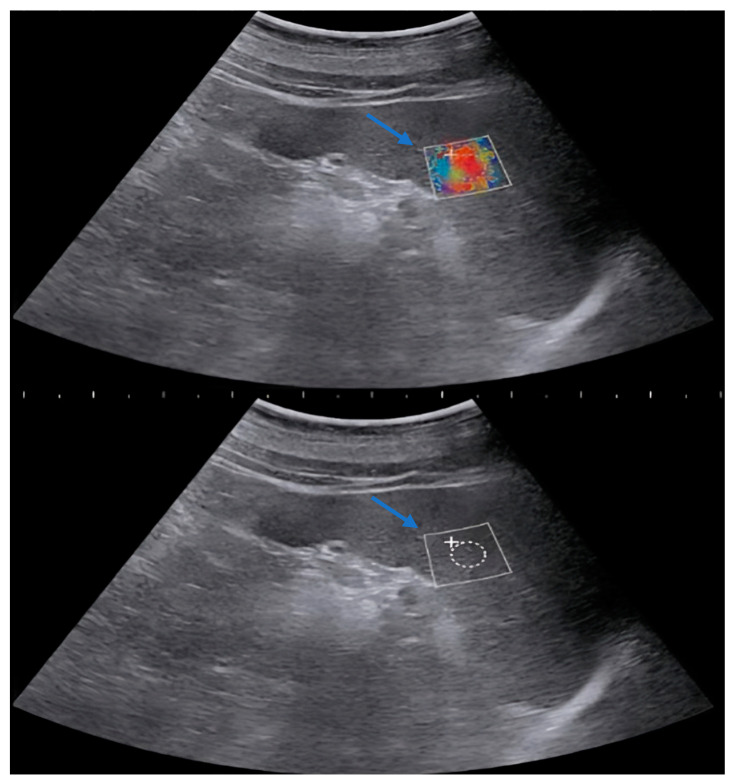
Splenic stiffness evaluation of 14-year-old boy with benign lymphoid hyperplasia (splenic craniocaudal length: 14 cm; stiffness on SWE: 13.36 kPa). Arrow: rectangular region of interest (ROI).

**Figure 2:**
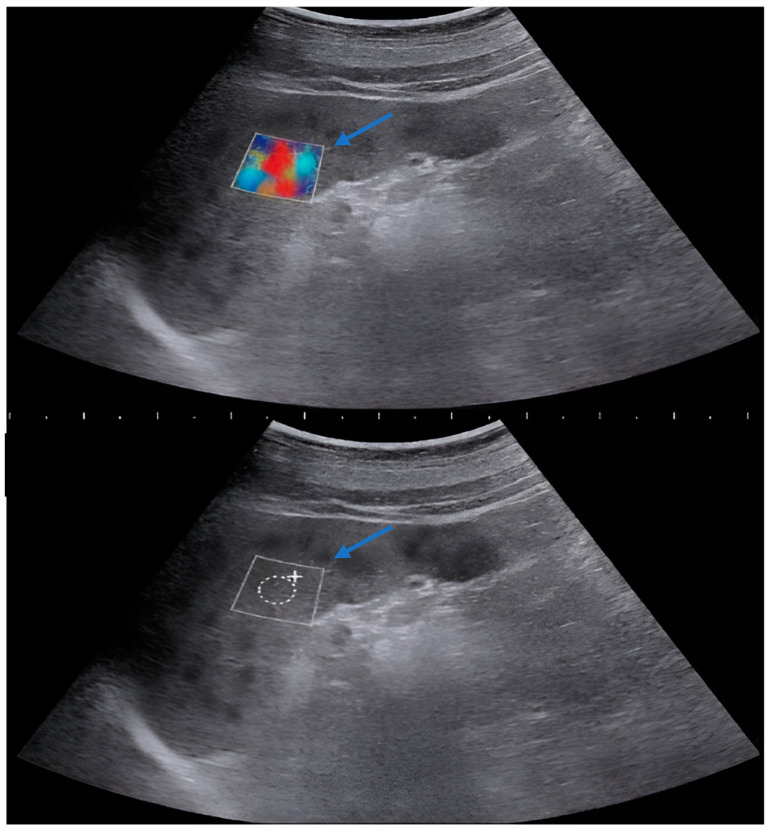
SWE imaging and assessment of 16-year-old boy with sickle cell anemia (splenic craniocaudal length 14.3 cm; stiffness on SWE: 14.05 kPa). Arrows: rectangular region of interest (ROI).

**Figure 3:**
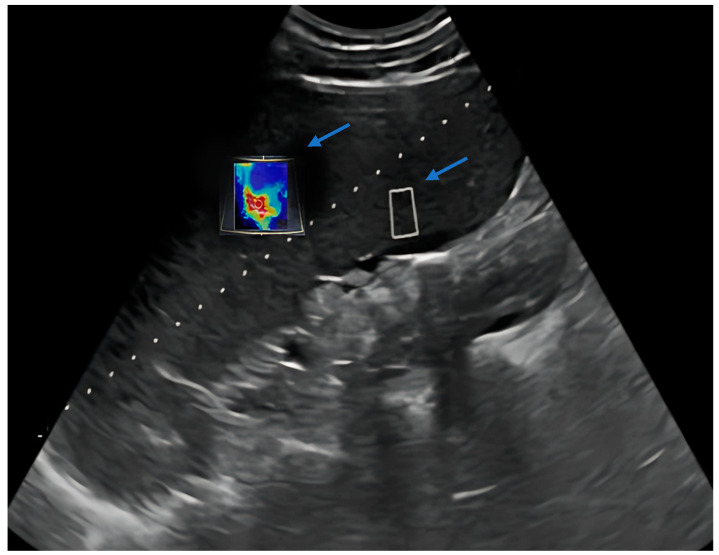
Splenic stiffness evaluation of 12-year-old girl with benign lymphoid hyperplasia (splenic craniocaudal length: 16.2 cm; stiffness on SWE: 13.27 kPa). Arrows: rectangular region of interest (ROI).

Measurements were performed by a single radiologist using a 2D-SWE (two-dimensional shear wave elastography measurements) feature-equipped US device (Siemens Acuson S3000 Ultrasound System HELX Evolution 12791052 Erlangen/Germany) with a 1–5 MHz convex probe. The spleen was imaged from a total of 10 different points, including the upper pole, lower pole, and midportion, using a 10 mm^2^ rectangular region of interest (ROI) after the patients were placed in the supine position and took deep inspiration (Figure 1, Figure 2 and Figure 3). Multiple measurements have been conducted at various levels to obtain a value that reflects the entire splenic parenchyma. The average of these measurements was recorded as splenic stiffness (kPa). The measured splenic dimensions and SWE-derived parenchymal stiffness were categorized according to the participants’ diagnoses. Subsequently, the mean splenic stiffness in each group was calculated and compared with the normal values within the same age group.

### 2.3. Statistical Analysis

The data were analyzed using the Statistical Package for Social Sciences (SPSS) for Windows 20 (SPSS Inc., Chicago, IL, USA). The normal distribution of data was assessed using the Kolmogorov–Smirnov test and Shapiro–Wilk test. Normally distributed numerical variables were presented as the mean ± standard deviation, while non-normally distributed variables were presented as minimum–maximum values. Categorical variables were presented as numbers and percentages. The comparison of mean age and gender differences between patients and the control group was conducted using an independent sample t-test and chi-square test, respectively. The stiffness of normal spleens and pathological spleens was compared using Student’s independent t-test. The degrees of stiffness in spleen enlargements secondary to different diseases were compared among themselves using Student’s independent *t*-test. A *p*-value < 0.05 was considered statistically significant.

## 3. Results

The study group included 1379 patients, of whom 892 (64.6%) were male and 487 (35.3%) were females, with a mean age of 10 years ± 2 months. The distribution of the patients into different pathology subgroups can be found in Table 2. The control group consisted of 1402 individuals, of whom 779 (55.5%) were male and 623 (44.4%) were female, with a mean age of 8 years ± 8 months. The mean age of the whole population was 9.27 ± 5 months. There was no difference between the mean ages of the study and control groups (*p* > 0.05). The pediatric population is defined in the literature as the age group ranging from 0 to 18 years. Our study population falls within the age range of 3 to 17 years, aligning closely with this definition, and there was no significant difference in the mean age between the groups.

The mean splenic craniocaudal dimension in the patient group was 14.17 ± 3.39 cm; meanwhile, it was 7.89 ± 1.53 cm in the control group. The mean splenic craniocaudal dimension was significantly higher in the patient group (*p* = 0.002). The mean splenic stiffness of the study group was 14.26 ± 1.33 kPa, which is significantly higher than that of the control group (13.56 ± 3.58 kPa, *p* = 0.03). No significant correlation was defined between splenic craniocaudal size and stiffness in both the control and study groups (*p* > 0.05).

In patients with splenomegaly, the highest quantitative values for splenic stiffness were observed in the portal hypertension group (16.63 ± 8.36 kPa), with significantly higher stiffness than both the control group (13.56 ± 2.56 kPa, *p* = 0.004) and the group with malignant infiltration (10.58 ± 4.77 kPa, *p* = 0.01). The mean splenic stiffness in the benign lymphoid hyperplasia group was higher than that in the control group, but this difference was not statistically significant (*p* = 0.63). Although the mean splenic stiffness in the benign lymphoid hyperplasia group (14.11 ± 4.14 kPa) was higher than that in the malignant infiltration group (10.58 ± 4.77 kPa), the difference was not statistically significant (*p* = 0.08). The mean splenic stiffness in the malignant infiltration group was significantly lower than that in both the control group (*p* = 0.005) and the group with splenic enlargement due to portal hypertension (*p* = 0.01).

In our study, patients with portal hypertension experienced the greatest increase in splenic stiffness. In the study group, the highest quantitative values for splenic stiffness were observed in the portal hypertension group, which was significantly higher than malignant infiltration, benign lymphoid infiltration subgroups, and the control group as well (*p* = 0.01). The mean splenic stiffness in the benign lymphoid hyperplasia group was similar to the control group (*p* = 0.09) and significantly higher than the malign infiltration subgroup (*p* = 0.003). Spleen stiffness was found to be significantly lower in the malignant infiltrative diseases group compared to the control group and the other study subgroups (*p* = 0.01) (Table 3).

## 4. Discussion

In our study, the mean splenic craniocaudal length in the patient population was calculated as 14.17 ± 3.39 cm, and it was observed to be statistically higher than that of the control group (*p* < 0.05). When the patients in the study group were subgrouped based on etiology, the highest splenic stiffness value was reached in the portal hypertension group with 16.63 ± 8.36 kPa. Although the stiffness degree in the benign lymphoid hyperplasia group was higher than that in the control group and malignant infiltration group with an average of 14.11 ± 4.14 kPa, it was not statistically significant (*p* > 0.05). Conversely, in the malignant infiltration group, the mean splenic stiffness was found to be significantly lower compared to other subgroups and the control group with 10.58 ± 4.77 kPa (*p* < 0.05). To the best of our knowledge, our study stands as the most extensive study in the literature, focusing on and comparing splenic stiffness in the pediatric population and the impact of various etiological factors on splenic stiffness.

The spleen is generally evaluated using ultrasonography as the primary imaging technique since ultrasound is not only reliable, easily accessible, and cost-effective, but it can also be used in real-time directly at the patient’s bedside. In addition, measuring the stiffness of organs and tissues via shear wave elastography (SWE) makes it a preferable method for clinicians. SWE is a novel technique employed to assess the rigidity of different tissues and organs, including the liver, breast, and thyroid. In recent years, there has been a growing use of SWE to non-invasively evaluate both normal parenchyma and lesions [32].

In recent years, numerous studies have been conducted using ultrasound elastography methods to investigate the relationship between liver fibrosis severity and spleen stiffness, especially in patients with portal hypertension. However, studies focusing on elastography examinations in patients with splenic enlargement due to causes other than portal hypertension have been limited in number and patient population. Additionally, most of the studies were conducted in the adult population. In this study, we aimed to investigate whether measuring spleen stiffness in a pediatric patient with spleen enlargement could contribute to the diagnosis [33,34]. We believe that our study serves as a guide for future, more comprehensive research in the assessment of splenic stiffness using non-invasive methods such as SWE in the differentiation of diseases affecting the spleen.

Bhatia A et al. demonstrated in their study on splenic stiffness in healthy children that there is no significant difference among age groups and genders. Furthermore, their study also revealed that the splenic stiffness values in healthy children ranged between 5.6 and 6.5 kPa [23]. Although our study did not show significant differences in the distribution among groups, a direct comparison between groups based on age and gender was not conducted. In addition, the mean spleen stiffness value in our control group was calculated as 13.56 ± 3.58 kPa, which is a higher value than the aforementioned study. We believe that this difference is due to the large population in our study. Additionally, the fact that the studies were conducted in different countries might also contribute to this situation. Further research is needed to establish advanced normative values on this matter.

Intrahepatic vascular resistance increases due to the development of fibrosis in the liver, leading to portal hypertension. Hypertension results in the development of collateral vessels and arterial vasodilatation, which increases blood flow to the spleen. An increase in spleen hardness is expected due to these mechanisms. However, splenomegaly in portal hypertension is not only due to congestion, but tissue hyperplasia and fibrosis also play an important role in this process. Studies in the literature show an increase in spleen hardness in portal hypertension [35,36,37]. Although the main aim of our study was not to investigate the relationship between liver fibrosis severity and spleen hardness, a significant increase in spleen hardness in portal hypertension was observed, which is consistent with other studies.

In another study by Bavu et al., the degree of liver fibrosis was evaluated using supersonic shear imaging, specifically in patients with hepatitis C [38]. In our study, similar to the elastographic values ranging from 7.73 kPa to 41.27 kPa with SWE in patients with portal hypertension-related splenomegaly, liver elastographic values also showed a wide range of values between 4.50 kPa and 33.96 kPa. In the comparison of ROC curves, it was observed that as the fibrosis level increased with supersonic shear imaging, the elastographic values increased for moderate and advanced fibrosis levels. The ROC curve value for the grade 2 fibrosis stage (2 F) was 0.846; the ROC curve value for grade 3 F was 0.857; and the ROC curve value for grade 4 F was 0.940. In this comparative study conducted using ROC curves for each fibrosis degree, it was observed that supersonic shear imaging, as an elastographic method, was significantly more effective as the fibrosis stage increased, and it was shown to be a non-invasive, fast, simple, and reliable method for evaluating liver fibrosis.

Hirooka et al. found that spleen stiffness could be a significant independent factor in the staging of fibrosis and cirrhosis as a non-invasive diagnostic assessment in cases confirmed by simultaneous liver biopsy in chronic liver patients. It was shown that elastographic values had significant diagnostic predictability in determining gastroesophageal varices in patients with advanced liver failure with necroinflammation showing as a result of biopsy and a hepatic venous pressure gradient above 12 mm Hg (*p* = 0.007). It was determined that measuring spleen stiffness with elastography alone or in combination with other non-invasive diagnostic tests could facilitate the non-invasive diagnosis of severe liver disease, enabling its application reliably and repeatedly in the diagnosis of esophageal varices [39]. In comparison with our study group with portal hypertension-related splenomegaly, an increase in elastographic values was observed between the patient and control groups based on the kPa parameter. In this study, patients with advanced liver fibrosis and esophageal varices were evaluated, and non-invasive tests that could help to classify cirrhotic patients into different risk categories were investigated. It was found that elastographic measurements correlated with HVPG, except for patients with vascular anomalies and portohepatic shunts, and spleen stiffness was a predictive assessment method for predicting OV and the prognosis of chronic liver disease.

All studies conducted on patients with portal hypertension due to chronic liver disease showed that SWE is the most effective elastographic examination compared to other modalities and reduces the need for repeated endoscopy and the measurement of hepatic portal venous gas in portal hypertension patients [8,35,36,40]. Furthermore, studies indicate that SWE could serve as an alternative to invasive methods for evaluating fibrosis in patients with portal hypertension and chronic liver disease. For these reasons, all patients included in our study were evaluated with SWE.

Transient elastography (TE) is an alternative technique utilized predominantly in the assessment of liver stiffness, measuring the speed of shear waves generated by vibrations. In the study that evaluated specific subgroups for causes of liver fibrosis, TE’s diagnostic accuracy for cirrhosis diagnosis was evaluated independently of underlying liver disease with a meta-analysis [14]. In our portal hypertension group, we observed an increase in elastographic values compared to the control group, which is consistent with the findings of this study. Additionally, correlating with other studies in the literature, the fibrosis stage increased proportionally with elastographic values. Furthermore, studies have shown that SWE could serve as an effective alternative to invasive methods for evaluating fibrosis in patients with portal hypertension and chronic liver disease [38,41].

In benign lymphoid hyperplasia of the spleen, an increase in the number and size of white pulp elements is observed due to increased lymphocytes, while the architecture of the spleen structures (red and white pulp) is preserved. In malignant infiltrative processes, this architecture is disrupted. Consistent with studies in the literature, our study demonstrated that spleen stiffness in benign lymphoid hyperplasia was lower than the group of portal hypertension and higher than the control group [20,24]. However, spleen elastography has not been evaluated in the literature for diseases with specific diagnoses in this group. There is a need for high case number studies to evaluate cases with specific diagnoses of splenomegaly due to lymphoid hyperplasia.

In our study, we observed that patients with splenomegaly due to malignant infiltration had the lowest stiffness values. Although there are not enough studies with a large number of cases in the literature on this subject, the decrease in spleen stiffness values may be due to the limited number of patients or necrosis developing in the spleen parenchyma. Meng et al. demonstrated that necrotic areas were observed in the spleen in 70% of 17 patients with splenic lymphoma [42]. Additionally, the coexistence of different diseases in the malignant infiltrative group reduces the reliability of our findings. More studies with a larger number of patients are needed to address this issue further.

Our study has several limitations. Firstly, the evaluation was performed by a single radiologist during our study; there was a lack of inter-observer agreement during the assessment as a major limitation, but we believe that this was mitigated by the high number of patients in our study. Furthermore, the retrospective nature of our study may introduce bias in patient selection. To mitigate this, we aimed to include as many eligible patients as possible who met the inclusion criteria. The patient’s follow-up data were not obtained. Therefore, we could not evaluate the possible correlation between the splenic stiffness value and the outcome of the diseases. The lack of numerical homogeneity among the groups in this study has been another limitation. Finally, the values of malignant infiltration or hyperplasia are not very different from the controls and do not help in the differential diagnosis. Further studies that concentrate on the non-invasive diagnosis of portal hypertension could offer promising results.

## 5. Conclusions

In conclusion, our study using the shear wave elastography method to examine splenic stiffness in pediatric patients has provided valuable insights and has positioned SWE as a hopeful additional tool for comprehensive evaluations of pediatric spleen-related diseases. Our findings highlight potential connections between the causes of diseases and changes in spleen stiffness. Therefore, we believe that SWE could be a promising and non-invasive tool for assessing the spleen in pediatric patients, yet further studies with supporting results are still needed.

## Figures and Tables

**Table 1:** Exclusion criteria and number of excluded patients from the study.

Exclusion Criteria	*n* (Excluded Patients)
History of acute splenic injury	12
Splenic Surgery	8
Previous treatment with non-selective beta-blockers	5
Shunt placement	4
Band ligation	1
Liver transplantation	3
Overt hepatic encephalopathy	3
İntrahepatic or extrahepatic malignancies other than lymphoid maligninant infiltration	9
Portal vein thrombosis or cavernous transformation	7
Presence of other chronic liver diseases	6

**Table 2:** Distribution of the patients in the study group.

	Male *n* (%)	Female *n* (%)	Total *n* (%)	Mean Splenic Size (cm)
Study group	892 (64.6)	487(35.3)	1379 (100)	14.17 ± 3.39
Malignant infiltration	126 (9.1)	67 (4.8)	193 (13.9)	
Portal hypertansion	213 (15.4)	144 (10.4)	357 (25.8)	
Benign lymphoid hyperplasia	553 (40.1)	276 (20)	829 (60.1)	
Control group	779 (55.5)	623 (44.4)	1402 (100)	7.89 ± 1.53

**Table 3:** Mean stiffness values according to groups.

Groups	Mean Splenic Stiffness Value
Control group	13.56 ± 3.58 kPa
Malignant infiltration	10.58 ± 1.18 kPa
Portal hypertansion	16.63 ± 7.73 kPa
Benign lymphoid hyperplasia	14.11 ± 3.91 kPa

## Data Availability

The datasets used and/or analyzed during the current study are available from the corresponding author on reasonable request.
